# Studies to Elucidate the Effects of Furostanol Glycosides from *Dioscorea deltoidea* Cell Culture in a Rat Model of Endothelial Dysfunction

**DOI:** 10.3390/molecules25010169

**Published:** 2019-12-31

**Authors:** Mikhail Korokin, Oleg Gudyrev, Vladimir Gureev, Liliya Korokina, Anna Peresypkina, Tatyana Pokrovskaia, Galina Lazareva, Vladislav Soldatov, Mariya Zatolokina, Mikhail Pokrovskii

**Affiliations:** 1 Department of Pharmacology and Clinical Pharmacology, Institute of medicine, Belgorod State National Research University, 308015 Belgorod, Russia; gudyrev@mail.ru (O.G.); produmen@yandex.ru (V.G.); pokrovskaia@bsu.edu.ru (T.P.); zinkfingers@gmail.com (V.S.); mpokrovsky@yandex.ru (M.P.); 2 Department of obstetrics and gynecology FPE, Kursk State Medical University, 305000 Kursk, Russia; lazarevaga@kursksmu.net; 3 Department of histology, embryology, cytology, Kursk State Medical University, 305000 Kursk, Russia; marika1212@mail.ru

**Keywords:** endothelial dysfunction, estrogens, Wistar rats, *Dioscorea deltoidea*, furostanol glucosides

## Abstract

Currently, there is no doubt surrounding a theory that the cardiotropic effects of sex hormones can be due to their direct effect on the cardiovascular system. In recent years, interest in the study of steroid glycosides has increased. We studied the effects of furostanol glycosides (protodioscin and deltozid) from the cell culture of the *Dioscorea deltoidea* (laboratory code DM-05) on the physiological and biochemical parameters of vascular endothelial function in hypoestrogen-induced endothelial dysfunction after bilateral ovariectomy. It was shown that the use of DM-05 at a dose of 1 mg/kg makes it possible to prevent the development of arterial hypertension (the level of systolic blood pressure (SBP) decreases by 9.7% (*p* < 0.05) and diastolic blood pressure (DBP) by 8.2%), to achieve a decrease in the coefficient of endothelial dysfunction by 1.75 times against the background of a hypoestrogenic state. With DM-05, an increase in the concentration of stable nitric oxide metabolites (NO_x_) by 45.6% (*p* < 0.05) and an increase in mRNA endothelial nitric oxide synthase (eNOS) expression by 34.8% (*p* < 0.05) was established, which indicates a positive effect of furostanol glycosides on the metabolism of nitric oxide after ovariectomy. Positive dynamics in the histological structure of the heart and the abdominal aorta indicate the pronounced endothelio- and atheroprotective effects of DM-05.

## 1. Introduction

Atherosclerotic cardiovascular disease (CVD) is the leading cause of morbidity and mortality in the world. According to the World Health Organization, there were 56.9 million deaths worldwide in 2016, and more than half of these (54%) were due to the top 10 causes of death. Ischemic heart disease and stroke are the world’s most significant killers, accounting for a combined 15.2 million deaths in 2016. These diseases have remained the leading causes of death globally in the last 15 years. Oxidative stress, endothelial activation, and endothelial dysfunction have been largely identified as the main alterations involved in the pathogenesis of macrovascular diseases [1]. The endothelium plays important roles in modulating vascular tone by synthesizing and releasing an array of endothelium-derived relaxing factors, including vasodilator prostaglandins, nitric oxide (NO), endothelium-dependent hyperpolarization (EDH) factors, and endothelium-derived contracting factors [2,3].

Currently, endothelial dysfunction is understood as an imbalance between mediators, which normally ensure the optimal course of all endothelium-dependent processes [4]. When endothelial dysfunction, production, interaction, and destruction of endothelial vasoactive factors are observed simultaneously with abnormal vascular reactivity, changes in the structure and growth of blood vessels are found to be associated with cardiovascular diseases [5,6].

The concept that the influence of sex steroids to one degree or another extends to the functional state of all organs and systems, including the cardiovascular system, is actively developing, and researchers are finding numerous confirmations. Sex differences in the development of cardiovascular diseases have been described in humans and animals. These differences explain the low incidence of cardiovascular diseases (stroke, hypertension, and atherosclerosis) in women during the reproductive period [7]. All sex differences in cardiovascular conditions have their basis in the combined expression of genetic and hormonal differences between women and men [8]. The functioning of the vascular endothelium can provide an idea of the gender differences in cardiovascular diseases, and of how and why the risk of cardiovascular disease changes dramatically with menopause. Since the increased risk of cardiovascular disease coincides with menopause, it is believed that female sex hormones, in particular estrogens, have endothelial as well as athero- and cardioprotective effects, which determines the possibility of the clinical use of natural and synthetic estrogens for the correction of cardiovascular diseases during menopause. In view of the above, it is obvious that an ample amount of attention is being paid to studying the possibility of the pharmacological correction of cardiovascular diseases and their complications in women during menopause by using estrogens as well as their synthetic and natural analogues [9,10,11,12].

In addition, there is evidence that phytoestrogens, even in a small amount, characteristic of the “Western” diet, have a cardioprotective effect. It was found that the habitual use of two phytoestrogens, isoflavone from soybeans and lignans from flaxseed, is associated with less rigidity of the aortic wall in postmenopausal women [13]. These compounds are used for the synthesis of hormonal drugs in the pharmaceutical industry. Furthermore, interest in steroid glycosides, as substances with a wide spectrum of biological estrogen effects on living organisms, is growing.

Our attention, which is focused on pharmacological effects in a model of endothelial dysfunction caused by bilateral ovariectomy, was inspired by a composition containing furostanol glycosides (protodioscin and deltoside in a ratio of 3/2) obtained using a super-producer strain of *Dioscorea deltoidea* cell culture (laboratory code DM-05).

The objective of this study is a pharmacological evaluation of the endotheliotropic effects of furostanol glycosides from *Dioscorea deltoidea* cell culture in a rat model of endothelial dysfunction after bilateral ovariectomy.

## 2. Results

### 2.1. Results of Vascular Endothelial Function Assessment

According to the study design, hypoestrogen-induced endothelial dysfunction was simulated by bilateral ovariectomy in female Wistar rats. The studied furostanol glycosides from the cell culture of *Dioscorea deltoidea* (DM-05) at a dose of 1 mg/kg were administered intraperitoneally once a day for 42 days. According to the protocol, the anesthetized animals were taken to the experiment on the 43rd day.

It was found that bilateral ovariectomy after 42 days leads to a statistically significant increase in systolic blood pressure (SBP) by 24.9% (*p* < 0.05) and diastolic blood pressure (DBP) by 30.5% (*p* < 0.05). At the same time, intraperitoneal administration of DM-05 at a dose of 1 mg/kg prevents the development of arterial hypertension; the level of SBP decreases by 9.7% (*p* < 0.05), and DBP decreases by 8.2% (Table 1).

**Figure 1:**
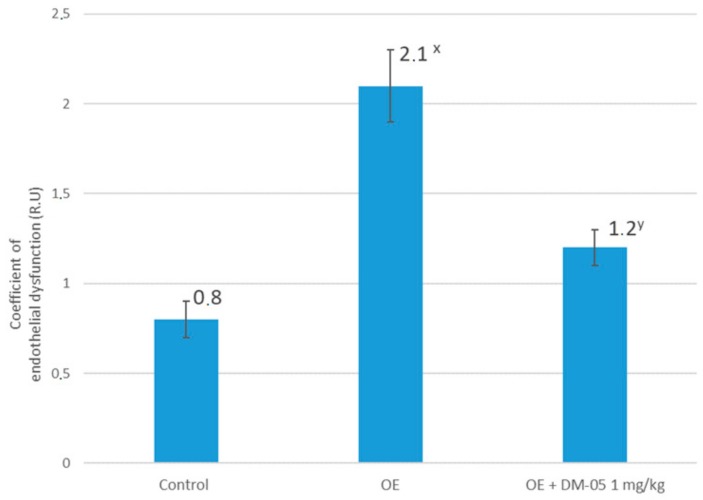
The values of the coefficient of endothelial dysfunction (relative units, RU) in animal groups with hypoestrogen-induced endothelial dysfunction, and its correction by furostanol glycosides from *Dioscorea deltoidea* cell culture DM-05 (*n* = 10). OE: group with bilateral ovariectomy; OE + DM-05: group with bilateral ovariectomy treated with DM-05 at a dose of 1 mg/kg; ^x^ *p* < 0.05 compared to the control; ^y^ *p* < 0.05 compared to the group with ovariectomy.

Figure 1 shows the calculated coefficient of endothelial dysfunction (CED), reflecting the results of functional tests for endothelium-dependent (EDVD) (acetylcholine 40 μg/kg iv) and endothelium-independent (ENVD) (nitroprusside 30 mg/kg iv) vasodilation in animals with hypoestrogen-induced pathology against the background of an administration of DM-05 at a dose of 1 mg/kg. In the control, CED was 0.8 ± 0.1 relative units (RU). Simulation of endothelial dysfunction by bilateral ovariectomy led to a statistically significant increase in CED to 2.1 ± 0.2 RU, and the intraperitoneal administration of DM-05 (1 mg/kg) led to a statistically significant decrease in CED to 1.2 ± 0.1 RU in comparison with the group with no treatment (Figure 1).

### 2.2. Results of the Evaluation of eNOS Expression and the Concentration of Stable Metabolites of Nitric Oxide (NO_x_)

The concentration of stable nitric oxide metabolites in endothelial dysfunction simulation by bilateral ovariectomy was statistically significantly reduced by 38.9% (*p* < 0.05), which reflects the development of endothelial dysfunction and change in the ratio of endothelium-dependent to endothelium-independent vasodilation. In the group with intraperitoneal administration of DM-05 within 42 days after bilateral ovariectomy, the concentration of stable metabolites of nitric oxide was significantly increased by 45.6% (*p* < 0.05), in comparison with the group with no treatment, and approached the NOx concentration in the control group (Table 2).

It was also found that the simulation of hypoestrogen-induced endothelial dysfunction does not lead to a significant change in eNOS expression (Table 2). Table 2 shows that intraperitoneal administration of DM-05 over 42 days leads to a statistically significant increase in mRNA eNOS expression by 34.8% (p < 0.05), in comparison with the group with no treatment after ovariectomy.

### 2.3. Results of the Histological Study

**Figure 2:**
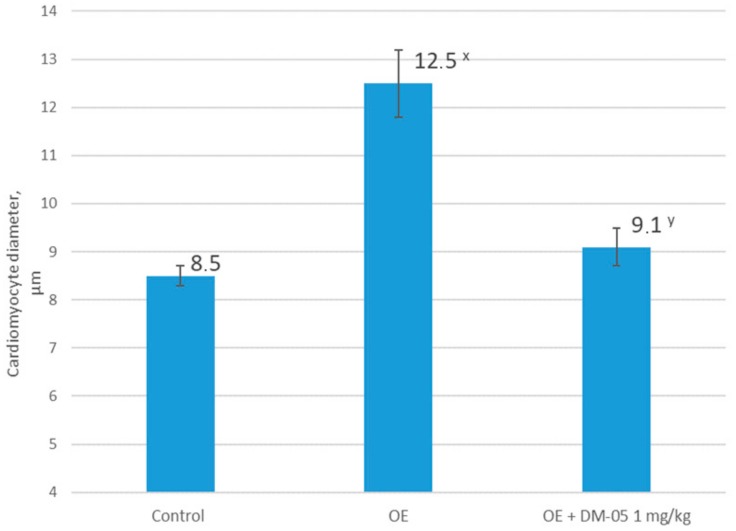
Cardiomyocyte diameter (µm) in animal groups with hypoestrogen-induced endothelial dysfunction and its correction by furostanol glycosides from *Dioscorea deltoidea* cell culture (DM-05) (*n* = 10). OE: group with bilateral ovariectomy; OE + DM-05: group with bilateral ovariectomy treated with DM-05 at a dose of 1 mg/kg; ^x^ *p* < 0.05 compared to the control; ^y^ *p* < 0.05 compared to the group with ovariectomy.

Hypertrophy of left ventricular cardiomyocytes up to 12.5 ± 0.7 (in the control 8.5 ± 0.21, µm), vascular changes in the form of a spastic state of the arterioles, and a thickening of their walls were noted in the heart (Figure 2). When stained with Rego hematoxylin, focal segmental or total lesions of a contracture type in the cardiomyocytes were detected.

The pronounced decrease in blood pressure and CED shown in this study was reflected in the morphological characteristics of the pathology—with the intraperitoneal administration of DM-05 (1 mg/kg), a statistically significant decrease in the cross-sectional diameter of cardiomyocytes was demonstrated (Figure 2).

The morphological study of histological sections of the wall of the abdominal aorta of the animals in the control group showed the preservation of the vessel architectonics. The intima was lined with a single layer of squamous epithelium or endothelium. Endotheliocytes were located along the edge of the intima on the basement membrane, one after another, flattened in shape. The cytoplasm of the cells was oxyphilic, and the nuclei were rod-shaped, located in the center of the cell and oriented longitudinally relative to the long axis of the cell.

**Figure 3:**
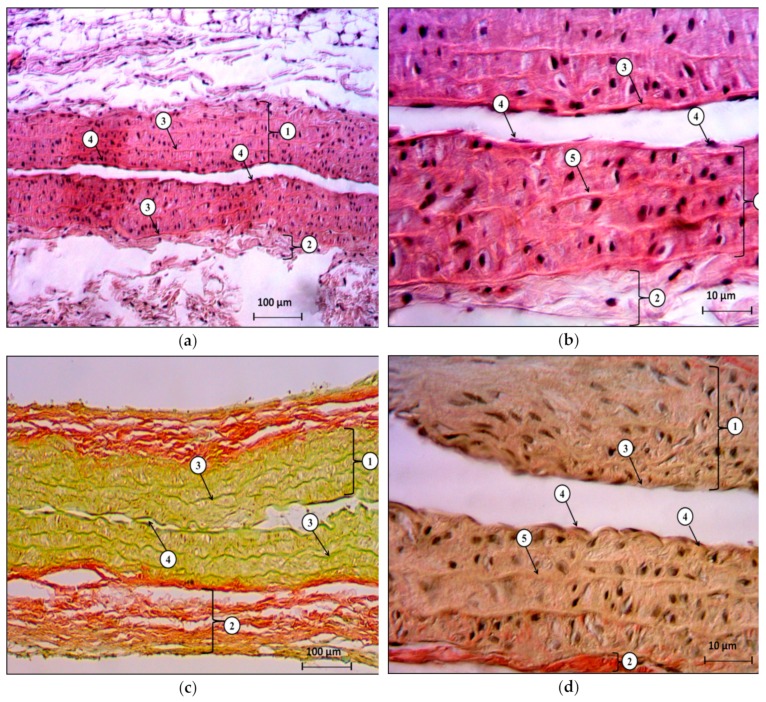
The histological structure of rat abdominal aorta in the control group. (**a**)—stained with hematoxylin and eosin, x40; (**b**)—stained with hematoxylin and eosin, x400; (**c**)—stained by Van Gieson’s method, x40; (**d**)—stained by Van Gieson’s method, x400. Designations: (**a**), (**c**): 1—middle shell; 2—outer shell; 3—fenestrated elastic membranes; 4—endotheliocytes located on the basement membrane. (**b**), (**d**): 1—middle shell; 2—outer shell; 3—inner shell; 4—endotheliocytes located on the basement membrane; 5—terminal elastic membranes.

In the media, brightly oxyphilic, sinusoidally shaped, fenestrated elastic membranes were well-visualized, and between these membranes, dark-basophilic cross-cut nuclei of circularly located smooth myocytes were defined. The cell density was low. In the outer shell (adventitia), loosely located collagen and elastic fibers were determined, and the fibroblastic cells were located between these fibers. There were no destructive changes, no squamous cell infiltration, no microthrombosis, and no signs of edema (Figure 3).

**Figure 4:**
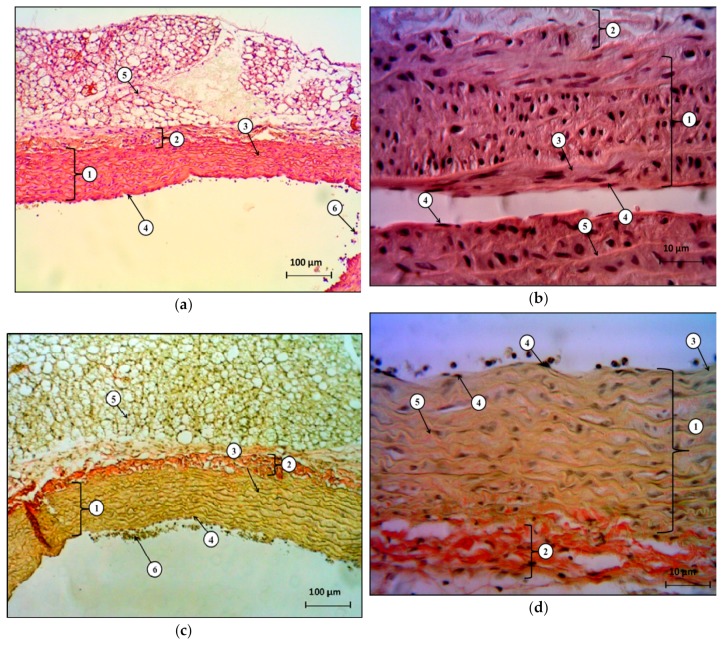
The histological structure of the abdominal aorta in the group with no treatment 43 days after bilateral ovariectomy: (**a**) stained with hematoxylin and eosin, x40; (**b**) stained with hematoxylin and eosin, x400; (**c**) stained by Van Gieson’s method, x40; (**d**) stained by Van Gieson’s method, x400. Designations: (**a**), (**c**): 1—middle shell; 2—outer shell; 3—graduated elastic membranes; 4—endotheliocytes located on the basement membrane; 5—paravasal tissue; 6—blood cells. (**b**), (**d**): 1—the middle shell; 2—the outer shell; 3—the inner shell; 4—endotheliocytes located on the basement membrane; 5—fenestrated elastic membranes.

In histological examination of the abdominal aorta, in the group with hypoestrogen-induced endothelial dysfunction, morphological changes were revealed, and these changes included signs of perivascular and pericellular edema, as well as inflammation of all the membranes of the vessel wall (aorta). Figure 4a,b show changes in the structure architectonics of the aortic wall. In the area of the intima, smooth myocytes are well-visualized (their nuclei are elongated, rod-shaped, and localized directly under the basement membrane of the endotheliocytes). There was also a thickening of the fenestrated elastic membranes determining the aorta elasticity. There were changes in the endothelium shape, as it appeared cubic.

When Van Gieson’s method was performed, signs of endothelial dysfunction were observed, and these were morphologically manifested in the fact that, in some parts of the intima, the intermittence of the endothelial layer was visualized, and lymphocytes were also visualized (Figure 4c,d). The detected marginal standing of leukocytes and the initial stages of thrombosis in the form of a cluster of blood cells in close proximity to the intima should be noted (Figure 4c).

The complex of changes in the heart and the abdominal aorta allows us to evaluate them as corresponding to a period of persistent increase in blood pressure with the development of vascular damage due to estrogen deficiency caused by bilateral ovariectomy in female Wistar rats.

**Figure 5:**
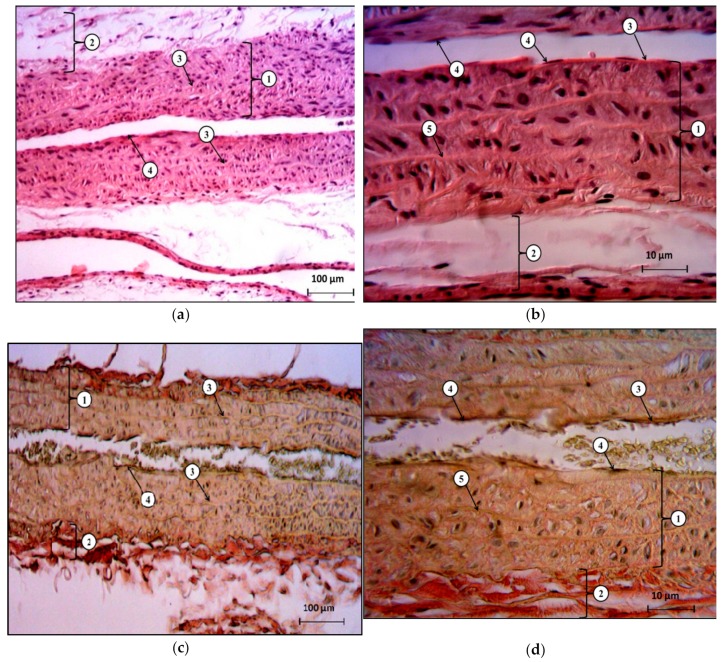
The histological structure of the abdominal aorta 43 days after bilateral ovariectomy in the group treated with 1 mg/kg DM-05: (**a**)—stained with hematoxylin and eosin, x40; (**b**)—stained with hematoxylin and eosin, x400; (**c**)—stained by Van Gieson’s method, x40; (**d**)—stained by Van Gieson’s method, x400. Designations: (**a**), (**c**): 1—middle shell; 2—outer shell; 3—fenestrated elastic membranes; 4—endotheliocytes located on the basement membrane. (**b**), (**d**): 1—the middle shell; 2—the outer shell; 3—the inner shell; 4—endotheliocytes located on the basement membrane; 5—fenestrated elastic membranes.

Amid administration of furostanol glycosides from DM-05 (1 mg/kg) for 42 days against the background of a hypoestrogenic state, pronounced positive dynamics of the histological structure of the abdominal aorta was found. In this group, the preservation of the architectonics of the layers of the vessel wall was found. The ratio of the thickness of the layers showed no visual differences in comparison with the data of the control group. Preservation of the endothelial lining, the location of the endotheliocytes in one layer on the basement membrane, a flat shape of the cells, and weakly oxyphilic cytoplasm were observed. The nuclei were rod-shaped, oriented along the blood vessel. No signs of pericellular and perivascular edema were detected. A slightly increased cell density per unit area of the section was observed, although without signs of destruction, which occurred in animals without pharmacological therapy after bilateral ovariectomy (Figure 5).

## 3. Discussion

According to some authors, surgical menopause is associated with a higher risk of vascular changes than natural menopause. In a study involving 144,260 postmenopausal women, premature menopause (compared to the absence of premature menopause) was associated with a significantly increased risk of developing and progressing coronary heart disease, heart failure, aortic stenosis, mitral regurgitation, atrial fibrillation, ischemic stroke, peripheral artery disease, and venous thromboembolism. For natural premature menopause, the risk ratio was 1.36; for surgical premature menopause, the risk ratio was 1.87 [14,15].

Sexual differences in decreased endothelial function are explained by changes in sex hormones with aging. In women, there is a progressive violation of endothelial function at all stages of menopause, which is partially associated with a decrease in estradiol level. The increased risk of cardiovascular disease in this group of women is primarily due to the reaction of tissues to a severe hypoestrogenic state in the absence of a physiological adaptation of the female body to new conditions [14,15].

In hypertension, the walls of great arteries become thicker and tougher, and so-called vascular remodeling processes occur [16]. In the study of arterial stiffness, these indicators were compared in men and women with essential hypertension relative to the level of blood pressure. This study demonstrated the presence of less vascular stiffness in premenopausal women and a convergence of indicators in men and postmenopausal women, which is probably associated with a loss of the protective effect of estrogen and, as a result, changes in the extracellular matrix of the arterial wall [13].

The prospective randomized Women’s Health Initiative (WHI) and the Early Versus Late Intervention Trial (ELITE) showed that starting menopausal hormone treatment (MHT) within 5 to 10 years of menopause is fundamental to the success of estrogen’s cardioprotection in postmenopausal women without adverse effects. Age stratification of the WHI data has shown that starting hormone treatment within the first decade after menopause is both safe and effective, and the long-term WHI follow-up studies are supportive of cardioprotection. This is especially true in estrogen-treated women who underwent surgical menopause. According to Naftolin et al., without continuing efforts at improving the drug regimens and removing harmful agents and practices from menopausal health care, it is inevitable that there will be increased aggregate misery, cost, and societal impact of age-related ovarian failure. The findings discussed above should be a call to action to further the search for effective lifestyle regimens, educational programs, diagnostic methods, and hormonal and non-hormonal agents with which the increased rate of CVD in postmenopausal years can be confronted [15].

In recent years, interest in steroid glycosides has increased, and research has been pursued in several directions. These compounds are used for the synthesis of hormonal medications in the pharmaceutical industry, and the interest in them as substances with a wide range of biological effects on living organisms is growing.

A component of steroid glycosides, sapogenins, serves as feedstock for the synthesis of steroid hormones and their analogues. The highest importance among these sapogenins, from which hormonal medications are obtained, is attributed to diosgenin, the leading position of which is due to the fact that the rhizomes of wild-growing and cultivated *Dioscorea* species with a high content of this steroid are used as raw materials for its production. In addition to *Dioscorea* rhizomes, agaves, various types of nightshade, fenugreek, yucca, and a number of other plants are used as industrial raw materials abroad.

The object of this study was to obtain a composition containing furostanol glycosides using a super-producer strain of *Dioscorea deltoidea* cell culture (laboratory code DM-05). A unique feature of this strain is the complete absence of spirostanol glycoside production in suspension culture, and the protodioscin and deltoside furostanol glycoside production in a ratio of 3/2, which is due to the absence of oligofurostanoside-specific alpha glycosidase in DM-05.

In the present study, we showed that bilateral ovariectomy in Wistar rats after 42 days leads to a significant increase in systolic and diastolic blood pressure, changes in the ratio of the response of endothelium-dependent to endothelium-independent vasodilation with a statistically significant increase in the coefficient of endothelial dysfunction by more than two times, compared to the control group. This, together with our data regarding the decrease in the concentration of final stable nitric oxide metabolites, and with the data regarding the change in the histological structure of the heart and the abdominal aorta, indicates a complex of functional, biochemical, and morphological changes associated with the progressive loss of endogenous estrogens 42 days after hypoestrogenic state simulation.

The results obtained in this study confirm our hypothesis about the effectiveness of furostanol glycosides in endothelial dysfunction and arterial hypertension after ovariectomy in rats. We showed that the intraperitoneal administration of DM-05 at a dose of 1 mg/kg prevents the development of arterial hypertension and eliminates the imbalance of endothelium-dependent and endothelium-independent vasodilation with a decrease in the CED in the hypoestrogenic state. At the same time, positive changes in the histological structure of the heart and the abdominal aorta indicate a pronounced endothelio- and atheroprotective effect of DM-05.

Understanding the biochemical pathways that regulate the metabolism and bioavailability of nitric oxide is necessary in assessing the effectiveness of the pharmacological correction of endothelial dysfunction [14]. To characterize the functioning of the nitroxidergic system in bilateral ovariectomy, we analyzed the concentration of stable nitric oxide metabolites as the final link of the L-arginine–eNOS–NO metabolic pathway and the expression of eNOS mRNA in the abdominal aortic homogenate.

The increase in NO_x_ concentration and the increase in the expression of eNOS mRNA in the use of furostanol glycosides from DM-05 indicate an effective correction of metabolic disorders of nitric oxide after ovariectomy.

An increase in eNOS expression helps to establish the equilibrium of transcriptional and post-transcriptional molecular mechanisms that belong to both proapoptotic and antiapoptotic pathways. In addition, the bioavailability of NO is determined not only by eNOS levels but also by the presence of substrate and eNOS cofactors, the phosphorylation status of eNOS, and the presence of reactive oxygen species (ROSs), which can inactivate eNOS [14]. The increase in eNOS mRNA expression when using furostanol glycosides might be due to the antioxidant activity of steroid glycosides. The antioxidant activity of furostanol glycosides is associated with the presence of a mobile hydrogen atom of the hemiketal hydroxyl group at C–22 and the formation of a stable intermediate inhibitor radical.

Based on the literature data and the results of this study, we think that the use of furostanol glycosides can slow down or prevent the formation of endothelial dysfunction and cardiovascular disease in postmenopausal women. To create a complete concept of the pharmacological activity of furostanol glycosides, the athero- and cardioprotective effects of DM-05 on models of cardiovascular diseases in estrogen deficiency require further detailed study. An important task of further research is the precise study of the pathways of DM-05′s influence on the content and metabolism of NO. DM-05 might affect the level of eNOS phosphorylation or the enzyme compartmentalization, increasing its activity.

## 4. Materials and Methods

### 4.1. Animals

The experiments were approved by the Belgorod State National Research University, Local Ethics Committee, Belgorod (Protocol #03/19). Ethical principles of handling laboratory animals were observed in accordance with the European Convention for the Protection of Vertebrate Animals Used for Experimental and Other Scientific Purposes, CETS No. 123. The animals were housed in an animal facility with a 12 h day/12 h night cycle and provided a standard laboratory diet and water. Experiments were carried out on 30 Wistar rats (three groups, 10 animals in each group) weighing 250 ± 25 g. For the study, the rats were taken with no external signs of disease, having passed the quarantine regime.

### 4.2. Experimental Design

The following groups were included in the experiment (10 animals in each group):

(1) control group—a group of falsely operated animals (control);
(2) a group with simulated endothelial dysfunction after bilateral ovariectomy (OE);
(3) a group with a correction of pathology with furostanol glycosides from a cell culture of *Dioscorea deltoidea* (OE + DM-05).

A false operation was performed on the animals of the control group under anesthesia (Zoletil^®^ 50 mg/kg of rat body weight, i.p.): the anterior abdominal wall was opened, and the wound was then sutured in layers without removing the ovaries. Animals of the control group were intraperitoneally injected with NaCl 0.9% 0.1 mL/100 g once a day for 42 days.

Endothelial dysfunction was simulated by bilateral ovariectomy in female Wistar rats. Bilateral ovariectomy was performed under general anesthesia (Zoletil^®^ 50 mg/kg of rat body weight, i.p.): the anterior abdominal wall was opened, the tube and peritoneal parts of the ovaries were ligated, and they were then removed [17]. Animals of this group were injected intraperitoneally with NaCl 0.9% 0.1 mL/100 g once a day for 42 days.

The composition of furostanol glycosides (protodioscin and deltoside in a ratio of 3/2) from a *Dioscorea deltoidea* cell culture under laboratory code DM-05 was administered intraperitoneally at a dose of 1 mg/kg once a day for 42 days starting from the day of surgery.

### 4.3. Assessment of Endothelial Dysfunction

On the 43rd day from the start of the experiment, in each animal under anesthesia (Zoletil^®^ 6 mg/100 g + Chloral hydrate 15 mg/100 g), the left carotid artery was catheterized for intravascular measurement SBP and DBP using the MP150 data acquisition and analysis system (Biopac Systems, Inc., Goleta, CA, USA). In continuous blood-pressure measurement mode, vascular tests of endothelium-dependent (acetylcholine 40 μg/kg) and endothelium-independent (sodium nitroprusside, 30 μg/kg) vasodilation were performed. Vasoactive agents were administered at 15 min intervals through a catheter inserted in the femoral vein. The level of endothelial dysfunction in the experimental animals and the level of its correction by the studied drugs were valued by CED expressed in relative units.

This coefficient was calculated by the following formula: CED = SBPNP/SBPAH, where SBPNP is the area of the triangle above the blood pressure (BP) recovery curve at a functional test with nitroprusside administration; SBPAH is the area of the triangle above the BP recovery curve at a functional test with acetylcholine administration. Points of the smaller side of this triangle include the point of BP before the test and the point of the maximum reduction of BP, and the longer side is the time of BP restoration [18,19,20].

After functional vascular tests, the animals were euthanized, a blood sample was taken from the left ventricle of the heart to determine the final stable nitric oxide metabolites, and the heart and abdominal aortic section were taken for histological studies, as well as for evaluation of eNOS gene mRNA expression.

### 4.4. Determination of eNOS Expression

Aortic tissue was harvested, homogenized, and incubated for 10 min at 37 °C in the “Extract RNA” solution. After sample lysing in the reagent, it was subjected to chloroform purification, and the resulting RNA precipitate was washed with isopropanol and 70% ethanol. The concentration of the resulting RNA was measured on an IMPLEN NanoPhotometer^®^ NP80 spectrophotometer (Implen GmbH, Munich, Germany). The output of the RNA was approximately 1000 ng/μL.

Reverse transcription was performed using the MMLV RT SK021 kit in accordance with the manufacturer’s protocol (Evrogen, Moscow, Russia). The mixture was gently mixed and heated at 70 °C for 2 min for the melting of RNA secondary structures and the subsequent annealing of the OligoDT primer (Evrogen, Moscow, Russia). After that, the samples were transferred to ice. The entire reaction mixture was incubated within 60 min at 40 °C in a T100™ ThermalCycler (Bio-Rad Laboratories, USA). To stop the reaction, the mixture was heated at 70 °C for 10 min. The resulting cDNA was diluted to a concentration of 1 ng/μL. The level of gene expression was evaluated relative to the values of the Gapdh reference gene.

### 4.5. Determination of Stable Nitric Oxide Metabolites

We used a modification of the method for determining stable NO metabolites, which allows one-step quantitative determination of total nitrates and nitrites after deproteinization of blood serum [21]. The principle of the method is the simultaneous reduction of nitrates to nitrites in the presence of vanadium chloride and a diazotization reaction with the subsequent development of a color, the intensity of which is determined spectrophotometrically at a wave length of 540 nm. Analysis of 100 μL of deproteinized serum was performed in 96 flat-bottom well plates. The sensitivity of the method on the Labsystems Multiskan MCC 340 (Thermo Fisher Scientific, Waltham, MA USA) is 1.7 μM. For colorimetric determination of the nitrite ion, Griss reagent was used, consisting of equal parts of Solution I (0.05% solution of *N*-naphthylethylenediamine in water) and Solution II (1% solution of sulfanilamide in 30% acetic acid). Both solutions were stored in the dark at 4 °C for several months. To prepare a solution of vanadium chloride, 400 mg of VCl_3_ was dissolved in 50 mL of 1 N HCl, followed by filtration through a paper filter. Freshly prepared solution was always used.

The level of NO metabolites (total concentration of nitrates and nitrites, NO_x_) was determined by the colorimetric method by the development of color in the diazotization reaction of sulfonamide nitrite, which is part of the Griss reagent. To construct the calibration curve, we used a 1 M solution of NaNO_2_ in water, which was stored at a temperature of −20 °C; before use, it was diluted 1000 times and a series of dilutions was prepared to construct a curve [21].

### 4.6. Histological Study

Samples of the heart and abdominal aorta were fixed in 10% formalin, followed by paraffin filling in an STP-120 carousel machine (Microm International GMbH, Germany). The blocks with the standard orientation of the pieces were poured at the station, so as to pour biological material into EC 350 paraffin (Microm International GMBH, Germany). To ensure standardization, paraffin filling was carried out in the form of multiblocks of 5–6 pieces. Slices for histological examination with a thickness of 5 μm were made on a semi-automatic rotary microtome with a system of transportation and a spreading of slices “NM 340E” (Microm International GMbH, Germany). Hematoxylin and eosin staining was carried out in a machine for staining histological sections and smears (Microm International GMbH, Germany). Descriptive histological studies were performed under an Axio Scope A1 microscope (Carl Zeiss Microimaging GMbH, Germany).

### 4.7. Statistical Analysis

For all data, descriptive statistics were used, and the data were checked for normal distribution. Distribution type was determined using the criterion of Shapiro–Wilk. In case of a normal distribution, the average value (M) and standard error of the mean (m) were calculated. In cases of abnormal distribution, the median (Me) and the quartile range (QR) were calculated. Between-group differences were analyzed by parametric (Student’s *t* criterion) or non-parametric (Mann–Whitney test) methods, depending on the type of distribution. Differences were determined at a 0.05 significance level. Statistical analyses were performed using Statistica 10.0 software [22].

## Figures and Tables

**Table 1:** The values of systolic (SBP) and diastolic blood pressure (DBP) in animal groups with hypoestrogen-induced endothelial dysfunction and its correction by furostanol glycosides from *Dioscorea deltoidea* cell culture (M ± m; *n* = 10), mmHg.

Experimental Groups	Systolic Blood Pressure	Diastolic Blood Pressure
Control	128.1 ± 6.0	95.7 ± 4.0
Ovariectomy	160.0 ± 6.2 ^x^	124.9 ± 5.5 ^x^
Ovariectomy + DM-05, 1 mg/kg	144.5 ± 5.9 ^xy^	114.9 ± 5.4 ^x^

^x^*p* < 0.05 compared to the control; ^y^ *p* < 0.05 compared to the group with ovariectomy.

**Table 2:** Values of NO_x_ and mRNA eNOS in animal groups with hypoestrogen-induced endothelial dysfunction and its correction by furostanol glycosides from *Dioscorea deltoidea* cell culture (M ± m; *n* = 10).

Experimental Groups	NO_x_ (mkM/mL)	mRNA eNOS (R.U)
Control	122.8 ± 11.6	2.7 ± 0.13
Ovariectomy	75.1 ± 8.4 ^x^	2.3 ± 0.21
Ovariectomy + DM-05, 1 mg/kg	109.4 ± 5.6 ^y^	3.1 ± 0.16 ^y^

^x^*p* < 0.05 compared to the control; ^y^ *p* < 0.05 compared to the group with ovariectomy.
